# Giant Myoepithelioma of the Soft Palate

**DOI:** 10.1155/2014/561259

**Published:** 2014-03-03

**Authors:** Murat Oktay, Huseyin Yaman, Abdullah Belada, Fahri Halit Besir, Ender Guclu

**Affiliations:** ^1^Department of Pathology, School of Medicine, Duzce University, 81620 Duzce, Turkey; ^2^Department of Otorhinolaryngology, School of Medicine, Duzce University, 81620 Duzce, Turkey; ^3^Department of Radiology, School of Medicine, Duzce University, 81620 Duzce, Turkey

## Abstract

Myoepitheliomas are benign salivary gland tumors and account for less than 1% of all salivary gland tumors. They are usually located in the parotid gland. The soft palate is very rare affected site. The differential diagnosis of myoepitheliomas should include reactive and neoplastic lesions. The treatment of myoepitheliomas is complete removal of the tumor. Herein, we report a case with giant myoepithelioma of the soft palate, reviewing the related literature.

## 1. Introduction

Myoepitheliomas are rare benign salivary gland tumors composed entirely or predominantly of myoepithelial cells. They account for less than 1% of all salivary gland tumors and are mainly located in the parotid gland and less often in the minor salivary gland of the oral cavity. Although extraparotid myoepitheliomas are extremely rare, they have occurred in the palate, submandibular gland, nasopharynx, nasal cavity, oral cavity, and cheek [1–7]. Myoepitheliomas frequently affect patients between the fourth and fifth decades of life without gender predominance [3, 4]. In this report, we present a case of large myoepithelioma arising in the soft palate.

## 2. Case Report

**Figure 1:**
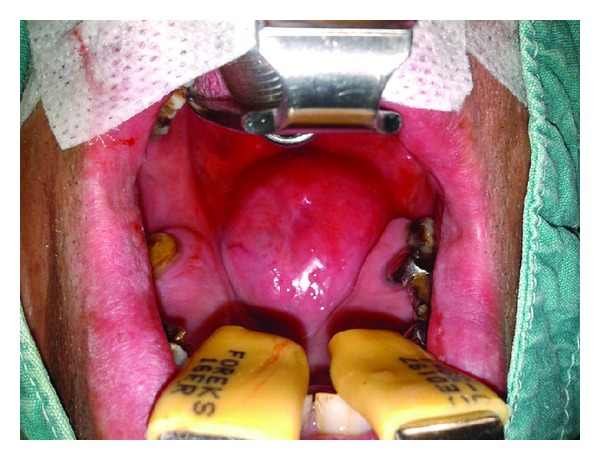
Preoperative view of large submucosal mass in the soft palate that nearly obstructs the nasopharyngeal port.

**Figure 2:**
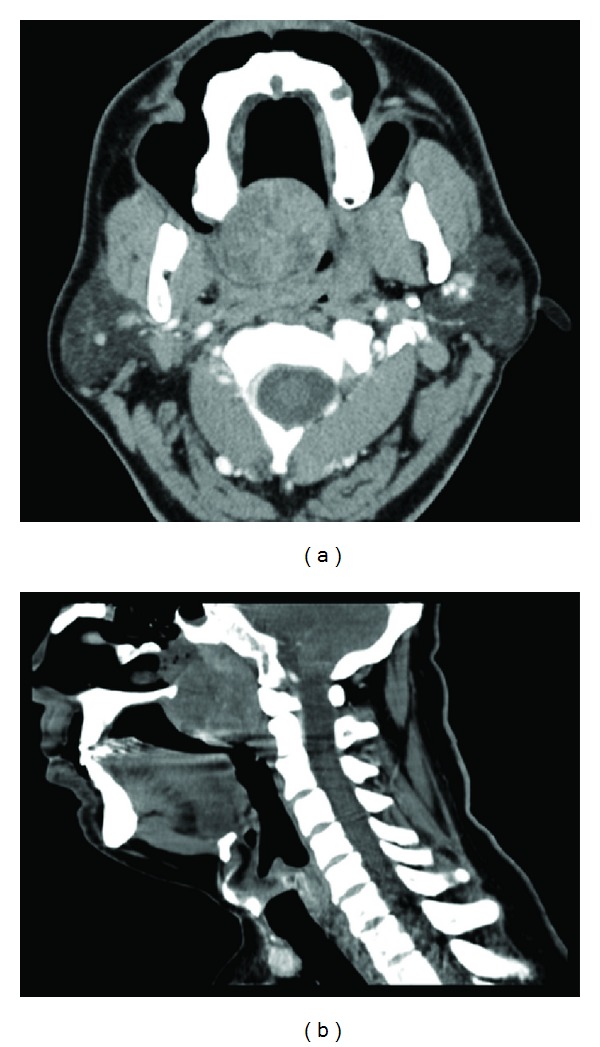
(a) Axial and (b) sagittal contrast-enhanced CT scan demonstrated a well-circumscribed, enhancing solid mass (5 × 3.5 cm) originating from the right posterior portion of the soft palate and enlarging toward the oronasopharyngeal airway space, but the lesion did not infiltrate adjacent fat plans and did not appear to involve the bone.

A 55-year-old male presented with a history of a painless swelling in his palate that gradually grew over the last five years. He complained snoring, obstructive sleep apnea, dysphagia, and hypernasal speech. There was a history of local trauma on his palate 11 years ago. Physical examination revealed, obstructing the nasopharyngeal port, a firm, immobile, well-circumscribed large submucosal mass in the soft palate (Figure 1). No lymph node was palpable in the neck. Computed tomography scan (CT) showed a well-defined, enhancing solid mass, measuring 50 × 35 mm, originating from the right posterior portion of the soft palate, but the lesion did not infiltrate adjacent fat plans and did not appear to involve the bone (Figure 2). Cytologic analysis of the aspirated material via a fine needle was composed of round to oval myoepithelial cells with eccentric nuclei and large eosinophilic cytoplasm.

**Figure 3:**
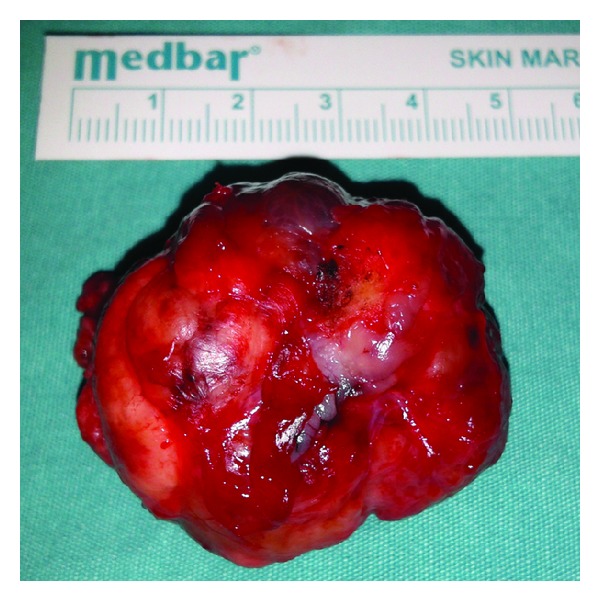
Postoperatively, lobulated, well-circumscribed tumoral specimen.

**Figure 4:**
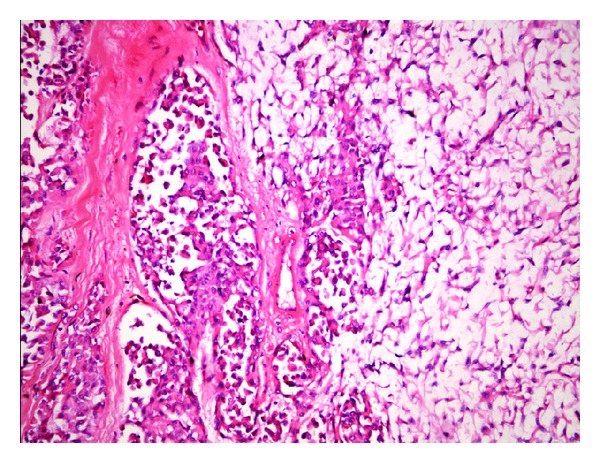
Nests and loose aggregates of tumor cells in a myxoid matrix (H-E, original magnification ×200).

**Figure 5:**
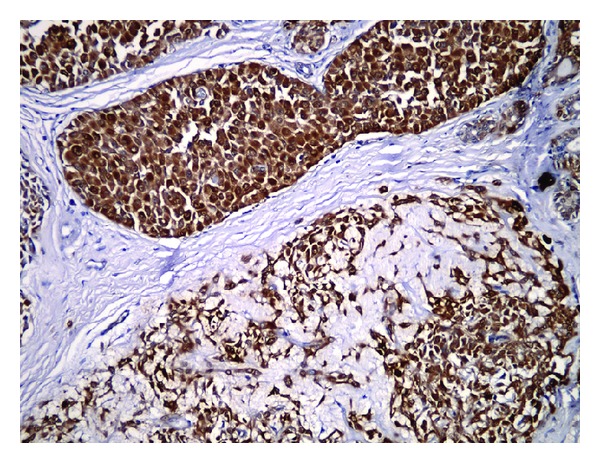
Tumor cells show reactivity with actin (×100).

The patient was operated via transoral approach under general anesthesia. The mass was totally removed with submucosal dissection. The surgical specimen was well-circumscribed, capsulated mass measuring 5 × 4 cm (Figure 3). On histopathological examination of the mass, the tumor was encapsulated and had plasmacytoid cells with homogeneous eosinophilic cytoplasm (Figure 4). Immunohistochemically, tumor cells stained with strong and diffuse positivity for S-100 protein, calponin, and smooth muscle actin (Figure 5). Histopathological diagnosis was myoepithelioma. Postoperatively, no complication was appeared such as infection, fistulae, or velopharyngeal insufficiency and no recurrence was seen during the 10-month follow-up period. Also, the speech of the patient has improved.

## 3. Discussion

We presented the case of huge, plasmacytoid type myoepithelioma which was localized in the oral cavity. Myoepitheliomas are slowly enlarging, asymptomatic, solid and well-circumscribed tumors, and usually less than 3 cm in diameter [8]. The palate is the most common location for myoepitheliomas in the oral cavity and is mainly located in the parotid gland and less often in the minor salivary gland of the oral cavity. Our case was 5 cm in size, solid well-circumscribed solid tumor localized in palate in the oral cavity.

CT and magnetic resonance scanning assist detection of the tumor location and also give information about structural properties and margin of tumor for the characterization of the tumor [9]. Histopathologically, they consist of spindle-shaped, plasmacytoid, clear, or epithelioid cell and the neoplastic cells are arranged in sheets, irregular collections, nests, interconnecting trabeculae, or ribbons. The spindle-cell variant is the most common subtype, presenting a proliferation of spindle-shaped cells and eosinophilic cytoplasm and forming a solid architectural pattern [2–5]. The plasmacytoid cell type has a predilection for the palate and presents round cells with eccentric nuclei and large and eosinophilic cytoplasm [2]. The plasmacytoid myoepitheliomas are immunoreactive for cytokeratin, vimentin, S-100 protein, and in some cases, muscle-specific actin and glial fibrillary acidic protein [2, 5]. The differential diagnosis of myoepitheliomas should include reactive and neoplastic lesions. Among these lesions are abscess, mucocele, schwannoma, neurofibroma, leiomyoma, benign fibrous histiocytoma, extramedullary plasmacytoma, rhabdomyosarcoma, smooth muscle neoplasms, pleomorphic adenoma, mucoepidermoid carcinoma, myoepithelial carcinoma, and other benign and malignant salivary gland neoplasms [2, 3, 5]. The distinction from pleomorphic adenoma is important because myoepithelioma is more aggressive than pleomorphic adenoma and occasionally transforms into malignant myoepithelioma [7]. Malignant myoepithelioma usually shows invasiveness, necrosis, and increased mitotic activity (>7 per 10 high-power fields). Although malignant myoepitheliomas are usually locally invasive and destructive, distant metastases are rare [10]. In our case, we confirmed the diagnosis with using CT imaging and the histopathological and immunohistochemical findings. We found noninvasive tumor, consisting of completely plasmacytoid myoepithelial cells which had nonmitotic activity.

The treatments of myoepitheliomas are superficial or total parotidectomy and surgical excision with tumor-free margins according to tumor location [2–6]. In our case, the tumor was located on the soft palate was removed completely with submucosal dissection.

## 4. Conclusion

Myoepitheliomas are benign and very rare salivary gland tumors in the soft palate and should be remembered in the differential diagnosis of the oral mucosa reactive and neoplastic masses. The treatments of myoepitheliomas are surgical excision. We recommended periodic control examinations and long-term followup postoperatively.
